# Identity reconstruction and digital addiction: the paradoxical mechanism of digital inclusion among the new generation of older adults

**DOI:** 10.3389/fpsyg.2026.1813587

**Published:** 2026-05-05

**Authors:** Haibei Chen, Xianglian Zhao, Qianjing Zhu

**Affiliations:** 1 School of Economics and Management, Taizhou University, Taizhou, Jiangsu, China; 2 School of Economics and Management, Nanjing University of Aeronautics and Astronautics, Nanjing, China

**Keywords:** digital addiction, digital inclusion, identity reconstruction, new generation of older adults, sustainable development

## Abstract

**Introduction:**

Against the backdrop of population aging and rapid digitalization in China, the new generation of older adults faces a growing paradox of digital inclusion. While digital participation can generate important benefits, it may also produce physical discomfort, health risks, psychological strain, and behavioral dependence.

**Methods:**

To achieve healthy and sustainable digital participation, this study develops an integrated framework drawing on dramaturgical, Motivation-Opportunity-Ability (MOA), and optimal distinctiveness theories and proposes a pathway linking digital inclusion, identity reconstruction, and digital addiction. Identity reconstruction is conceptualized as a five-stage process comprising identity formulation, identification, dissemination, co-construction, and maintenance. Then algorithmic perception, platform pressure, and digital reflection are further introduced as moderating variables. Using a mixed-methods design that combines structural equation modeling and fuzzy-set qualitative comparative analysis, this study validates the proposed framework and identifies three patterns of digital addiction: identity operation, identity reinforcement, and norm-guided.

**Results:**

The findings indicate that digital inclusion does not directly trigger digital addiction but indirectly influences sustained use through multiple stages of identity reconstruction. Moreover, algorithmic perception, platform pressure, and digital reflection exert differentiated moderating effects on the relationship between identity reconstruction and digital addiction. The three identified patterns move beyond conventional explanations based on limited digital skills, cognitive decline, or insufficient self-control, and instead reveal the central role of differentiated identity mechanisms in the formation of digital addiction.

**Discussion:**

These findings advance our understanding of the unintended consequences of digital inclusion and point to the need for multilevel interventions that better align identity needs, platform structures, and social support.

## 1 Introduction

### 1.1 Research background

The structure of 21st-century society is being profoundly reshaped by two irreversible macro trends: the ongoing acceleration of population aging on a global scale and the digital technology revolution centered on the Internet, big data, and artificial intelligence. These two forces intertwine and reinforce each other, producing systemic impacts on social operations, economic structures, public governance, and forms of individual life. From the perspective of population structure, most countries worldwide are entering an aging society at an unprecedented pace. The continuous expansion of the elderly population means that their proportion and influence in society are steadily increasing. Simultaneously, the rapid development of digital technology is reconstructing modes of information dissemination, patterns of social interaction, and the logic of economic operation, becoming deeply embedded in all aspects of everyday human life and serving as the infrastructure for modern society. The widespread adoption of digital technology has incorporated people of all ages into digital governance and service systems, and society’s overall dependence on digital technology continues to grow (Bucci et al., 2019).

Against this global backdrop, China displays particularly unique structural characteristics, simultaneously experiencing the world’s largest and fastest process of population aging and one of the most widespread and profound social digital transformations, all within the same historical period. According to the 56th Statistical Report on China’s Internet Development released by the China Internet Network Information Center, as of June 2025, the number of monthly active “silver-haired” Internet users aged 60 and above in China reached 161 million, accounting for 52.0% of the total elderly population. This data indicates that the elderly group is significantly participating in and sharing the benefits of digital development. Unlike many countries, where the elderly population skews older, China’s senior population is relatively younger, with a higher proportion of younger and healthier elderly individuals. This group is not only expanding rapidly in size but also stands out in terms of economic capability, willingness to participate socially, and consumption potential, gradually becoming an important social group worthy of attention. Related research and market reports have begun to use terms such as new generation elderly or emergence of the new elderly to describe them. The 2017 China Elderly Consumption Habits White Paper defines the new elderly as those aged 61–76 years with relatively stable sources of income. Unlike the traditional image of older adults as a vulnerable group, the new generation of older adults often has a higher level of education, greater economic independence, and richer professional experience. More importantly, this group grow up during a period of rapid societal transformation and technological change; in both their work and daily lives, they have been exposed to and used digital technologies to varying degrees (Chee, 2024), possessing a certain foundation of digital literacy and experience with technology adoption. They are more receptive to new things and have a stronger desire for social participation, actively and autonomously integrating into the digital social life. Therefore, the new generation of older adults is different from traditional elderly groups and cannot simply be equated with digitally disadvantaged groups. Their digital inclusion process exhibits more complex and multidimensional characteristics.

The digital inclusion of the new generation of older adults generally refers to their ability to effectively access and skillfully use digital technologies, products, and services, actively participate in digital social activities, and thereby meet the growing needs for elderly care services and personal development in later life (Lythreatis et al., 2022). To some extent, digital inclusion breaks through the limitations of time and space, helping the new generation of older adults to rebuild their social networks, expand channels for obtaining information, and enrich their spiritual and cultural lives. Consequently, their later years have become more open, diverse, and dynamic. Although digital inclusion brings the new generation of older adults an immersive user experience and considerable digital dividends, it may also lead to digital addiction, which can result in physical discomfort, health problems, psychological distress, and behavioral dependency (Demir et al., 2025). Excessive use of digital technology may not only weaken the original positive effects of digital technologies but also partially offset their roles in social integration and life improvement, creating a complex phenomenon in which digital empowerment and negative backlash coexist for elderly users. Digital addiction is also known as technology, Internet, or computer addiction. It was initially viewed as a type of non-chemical addiction based on human-computer interaction (Çimke et al., 2023). Although the academic community has yet to reach a unified consensus on its conceptual definition and diagnostic criteria, it is generally recognized that digital addiction manifests as the irrational, excessive, and uncontrolled use of digital platforms or devices. This means that individuals continue to depend on digital media even in the absence of strong functional needs and find it difficult to satisfy the same psychological or social needs through alternative means (Khang et al., 2013). Related research has pointed out that the development of digital addiction may be closely related to various factors, including individual levels of digital literacy, self-need structure, differences in income and resources, interpersonal relationships, sunk costs, as well as platform algorithms and commercial manipulation mechanisms (Almourad et al., 2020; Jeong and Bae, 2022; Kwon et al., 2016).

The new generation of older adults exhibits unique characteristics that distinguish them from older digital immigrants and digital refugees. On the one hand, they possess the motivation and foundation to actively integrate into the digital society. On the other hand, they are also more susceptible, under the combined effects of algorithmic recommendations, platform stickiness design, and commercial logic, to falling into usage dilemmas that are difficult to perceive and self-regulate (Yao, 2025). Compared with other age groups, digital addiction among the new generation of older adults is often more closely linked to psychosocial factors such as loneliness, social isolation, role transitions, and a lack of emotional support. Based on this, focusing on this group of new generation older adults who have unique historical experiences, backgrounds of social role transformation, and distinct patterns of digital behavior, and conducting specialized research on their digital inclusion and digital addiction not only helps to enhance the precision and explanatory power of related research but also holds significant contemporary relevance and practical value. Although in recent years, the issue of digital addiction among older adults has gradually entered the academic spotlight and relevant research is accumulating, overall, existing studies mainly remain at the level of describing the superficial phenomena of digital addiction and empirically analyzing its influencing factors and causes. Most research tends to view older adults as a relatively homogeneous group or only distinguishes them based on external characteristics such as age stage or type of residence, primarily explaining their situations through individual traits, social support networks, or access to structural resources (Freak-Poli et al., 2021). Within this research framework, the digital behavior of older people is often understood as a passive adaptation to the technological environment or a reaction to external conditions, with less in-depth exploration of how, in the process of digital inclusion, they reinterpret their roles, reshape their social positions, and reconstruct their self-identity. To address these shortcomings, this study introduces an analytical perspective on identity reconstruction, focusing on the proactive practices of the new generation of older adults during digital inclusion, aiming to answer the core question of how they reinvent themselves. Identity reconstruction is not simply a matter of technological adaptation; rather, it is a series of psychological and behavioral adjustment processes by which a new generation of older adults, after experiencing significant changes in their social roles, redefine themselves and rebuild their sense of social belonging and value. In this study, identity reconstruction specifically refers to the conscious and purposeful use of digital platforms and media by the new generation of older adults to redefine and project their self-image, actively challenge and correct traditional social stereotypes about the elderly, and construct a more self-affirming, positive, diverse, and modern elderly identity to compensate for the loss of identity resulting from retirement. Unlike a few studies that merely point out the paradox of digital inclusion, this study not only pays attention to the existence of such paradoxes but also seeks to reveal the mechanisms behind their formation and transformation pathways, thus exploring possible solutions to break them.

Therefore, this study aims to systematically uncover the internal mechanisms of the digital inclusion paradox among the new generation of older adults and, based on this, propose targeted interventions and support strategies to help them achieve healthy digital inclusion. To achieve this goal, the study will focus on the following research questions: First, what specific manifestations does identity reconstruction take in the process of digital inclusion among the new generation of older adults, and what are the underlying motivations? Second, how and why is the process of identity reconstruction associated with digital addiction? Third, which factors play key catalytic or inhibitory roles in this transformation process? Fourth, how can a multilevel intervention and support system be constructed to effectively resolve the digital inclusion paradox and guide the new generation of older adults toward sustainable and healthy digital inclusion?

### 1.2 Motivation and contribution

To systematically reveal the internal mechanisms underlying the coexistence of identity reconstruction and digital addiction among the new generation of older adults during their digital inclusion, this study integrates dramaturgical, Motivation-Opportunity-Ability (MOA), and optimal distinctiveness theories to construct a comprehensive framework. This framework can simultaneously address digital inclusion behaviors, identity reconstruction logic, and digital addiction risks specific to the new generation of older adults. On this basis, the study proposes the core pathway of “digital inclusion-identity reconstruction-digital addiction”, elucidating how digital inclusion profoundly influences the digital behaviors of the new generation of older adults through identity reconstruction. Identity reconstruction is divided into five progressive stages: identity formation, identification, dissemination, co-construction, and maintenance. This systematically depicts the dynamic evolution of the new generation of older adults in the process of digital inclusion, from self-positioning and identity confirmation to sustained interaction and identity consolidation in the digital world. Simultaneously, three key moderating variables, algorithmic perception, platform pressure, and digital reflection, are introduced to outline the differentiated pathways through which identity reconstruction affects digital addiction under different circumstances. Based on the above theoretical analysis, a theoretical model is constructed to illustrate how digital inclusion among the new generation of older adults influences digital addiction through multistage identity reconstruction. Finally, a mixed research approach combining SEM and fsQCA is used to test the rationality of the theoretical model, and to further analyze multiple pathways of digital addiction under different combinations of influencing factors. Through this research design, this study aims to explain the paradoxical mechanism of digital inclusion while providing theoretical foundations and practical insights to guide the new generation of older adults toward healthy and sustainable digital inclusion in the future.

This study offers a multidimensional analytical framework by integrating these theoretical and empirical analyses. Its primary contributions are outlined as follows: (1) By introducing the perspective of identity reconstruction, this study systematically reveals how the new generation of older adults influences the consequences of their digital usage behaviors through identity construction in the process of digital inclusion. By incorporating digital inclusion, identity reconstruction, and digital addiction into a unified analytical framework, this study deepens the understanding of the paradox of digital inclusion among older adults from an internal mechanism perspective, enriching the theoretical explanatory pathways of behavioral research on older adults in the digital social context. (2) The analysis shifts its focus from traditionally defined older adults to a new generation of seniors who possess unique life trajectories and digital experiences, thus avoiding the tendency to oversimplify and homogenize the elderly. By focusing on their digital practices and processes of identity adjustment in the context of changing social roles after retirement, this study uncovers the unique mechanisms by which digital inclusion and digital addiction develop among the new generation of older adults, thereby enhancing the contemporaneity and interpretative power of related research. (3) This study reveals multiple pathways to the formation of digital addiction under different combinations of conditions, providing a more flexible analytical tool for understanding the complexity of digital behavior among older adults. Based on this, targeted recommendations are proposed, offering empirical evidence to guide the new generation of older adults toward healthy and sustainable digital inclusion.

The remainder of this paper is organized as follows. The following section briefly discusses the related literature and innovations. Section 3 is devoted to related theories and the paradoxical mechanism of digital inclusion. Section 4 includes the method of selection, questionnaire design, sample selection, and data collection. Section 5 provides the basic processing and detailed analysis of the results. Finally, Section 6 summarizes the conclusions, outlines the limitations of the study, and presents possible directions for future research.

## 2 Literature review

### 2.1 Research on digital inclusion

Current research generally holds that digital inclusion among older adults is not the result of a single factor, but rather a complex process involving the interplay of multiple elements such as individual cognitive and psychological characteristics, technological and environmental conditions, as well as social and family support (Yu et al., 2024; Zhao et al., 2021). At the individual level, digital literacy and cognitive ability are widely regarded as fundamental factors influencing digital inclusion among older adults. Studies have shown that older adults often lack proficiency in digital skills, information screening, and working memory, which limits their understanding, learning, and continued use of digital technologies (Petrovcic et al., 2023). At the same time, age-related changes in cognitive function such as memory decline, reduced attentional allocation, and weakened executive function tend to increase their difficulty in adapting to digital environments, thereby raising the risk of digital exclusion (Li and Kostka, 2024). Additionally, demographic characteristics such as educational attainment, income level, and area of residence exhibit significant differences in digital inclusion for older populations; higher levels of education and better economic status generally contribute to a greater willingness and actual ability to adopt digital technologies (Fisk et al., 2023). In addition to objective capabilities, individual subjective psychology and cognitive orientations play a key role in the process of digital inclusion. Relevant studies indicate that attitudes toward technology, self-efficacy, learning motivation, and perceptions of digital risk directly influence the degree to which older adults accept and use digital technologies (Zhu et al., 2025). Positive attitudes toward technology and higher self-efficacy help boost their confidence in learning and willingness to use these tools, while concerns about operational failure, privacy breaches, or online scams may intensify avoidance behaviors and lower digital engagement. Some studies have further emphasized that higher levels of digital health literacy and ongoing cognitive engagement help older adults maintain stable technological proficiency in dynamic digital environments, thereby supporting long-term digital inclusion (Ma et al., 2022). At the technological and environmental levels, the design features of digital products and services are seen as important external conditions affecting digital inclusion among older adults. Existing research has shown that overly complex interfaces, interaction logic inconsistent with the cognitive habits of seniors, and insufficient age-friendly design are key factors hindering their continued use of digital technologies (Carney and Kandt, 2022). In contrast, simple and intuitive interface layouts, clear and precise feedback, and customizable functions can effectively lower the threshold for technology use and improve the digital experience of older adults. Furthermore, stable Internet infrastructure and a secure, reliable digital environment are crucial prerequisites for ensuring that older adults can smoothly participate in digital society. From a social structure perspective, social and family support play an empowering role in the digital inclusion of older adults that cannot be overlooked. Research shows that support from community organizations, peer networks, and social institutions can provide emotional, informational, and instrumental resources, alleviating the digital anxiety and technological adaptation pressures faced by the elderly, and enhancing their motivation and confidence in learning about and using digital technologies (Ferreira et al., 2016). Simultaneously, family support, as a form of micro-level social support embedded in daily life, has unique advantages for older adults in acquiring digital skills (Bossio and McCosker, 2021). Intergenerational support from family and friends, especially from younger generations during company, demonstration, and patient guidance, not only helps reduce technological anxiety but also strengthens operational abilities and technological familiarity, promoting the continued application of digital technologies in everyday life. Collectively, these factors influence the learning trajectory, depth of use, and breadth of participation in digital inclusion among older adults.

Most existing studies tend to define older adults as a marginalized or disadvantaged group within the digital society, often focusing their research perspectives on issues such as digital exclusion, lack of ability, or structural inequality, thereby exploring the obstacles and dilemmas faced by older adults in terms of digital inclusion (Zhao and Zhu, 2024). However, this predominantly disadvantaged-compensation approach easily overlooks the significant heterogeneity within the older population and makes it difficult to fully reveal the complex consequences of digital inclusion, thus, to some extent, obscuring the existence of the paradoxes inherent in it. Digital inclusion does not improve older adults’ social circumstances in a one-way manner; its impact can manifest in enhanced abilities and expanded social participation, but may also introduce new pressures, divisions, and the reproduction of inequalities. In terms of its effects, digital inclusion not only reshapes the individual capacity structure of older people but also, to a certain extent, broadens their social roles and spheres of action (Olsson and Viscovi, 2020). On the one hand, learning and using digital technologies helps enhance older adults’ abilities to acquire information, solve problems, and engage socially, promoting a shift from passive adaptation to active participation. On the other hand, the development of digital platforms has offered some older adults new opportunities for work and participation, such as flexible employment, online services, and experience-based labor, thereby increasing their willingness to participate in the workforce and changing the traditional trajectory of complete withdrawal from social life after retirement.

It is especially noteworthy that the new generation of older adults, as an important group distinct from the traditional elderly population studied in the past, exhibits significant differences in digital behavior and social roles. Compared with previous elderly groups who passively accepted digital technology, the new generation of older adults often possesses higher educational levels, richer professional experience, and a more proactive attitude toward using technology. They tend to actively integrate digital technology into their daily lives and exert influence over their families, communities, and even online spaces. This group is no longer merely recipients in the process of digital inclusion; they are gradually becoming participants, disseminators, and co-creators of digital practices. In this context, the process of digital inclusion for the new generation of older adults is essentially a continuous resocialization process. Through ongoing learning, adaptation, and self-renewal, older adults can reconstruct their self-identity, expand their social connections, and achieve value continuation within a digital environment. However, this process is accompanied by increased competency requirements, changing role expectations, and heightened pressures of responsibility, all of which highlight the duality and complexity of digital inclusion. Therefore, it is necessary to move beyond the simplistic research paradigm that labels older adults as vulnerable groups. Instead, we should examine the practical paths taken by different generations of older adults in digital inclusion and the underlying structural paradoxes from dynamic, multidimensional, and differentiated perspectives. These issues form key theoretical and practical topics that future research must address.

### 2.2 Research on digital addiction

Digital addiction is not a single concept but rather encompasses various forms of expression, such as Internet addiction, gaming addiction, Internet compulsion, and information anxiety. Its causes are often closely linked to the high accessibility and continuous stimulation characteristics of digital devices, platforms, and media (Meng et al., 2022). Research shows that digital addiction has significant negative effects on individuals at multiple levels, mainly reflected in physical and mental health, work performance, social relationships, and life satisfaction. First, in terms of physical and mental health, the impact of digital addiction involves both physical and psychological dimensions. On the one hand, prolonged and excessive use of digital devices can easily lead to physical health problems such as deteriorating eyesight, impaired sleep quality, and sedentary-related illnesses. On the other hand, continuous, high-intensity informational stimulation and dependence on digital media can also increase psychological burden, triggering or exacerbating negative emotional states such as anxiety, depression, anger, and sadness (Foroughi et al., 2022). Second, in terms of work performance, digital addiction often weakens an individual’s attention control ability, making them more susceptible to distractions from digital content during work or task execution. This reduces concentration and work efficiency, ultimately adversely affecting overall job performance (Moqbel and Kock, 2018). Thirdly, on the level of social relationships, excessive reliance on social interaction within virtual environments may result in a reduction of face-to-face communication in the real world, weakening real-life social connections, and thereby impacting the quality of interpersonal relationships, even leading to an imbalance between humans and technology (Meshi et al., 2020). Finally, from the perspective of life satisfaction, the original intention of integrating digital technologies was to enhance convenience and the overall quality of life. However, when digital usage lacks reasonable boundaries and evolves into addictive behavior, the accompanying stress, sense of loss of control, and negative experiences may actually erode subjective well-being and lower overall satisfaction with life.

Existing research often regards digital addiction as a pathological behavior, emphasizing its core characteristics of compulsive and uncontrolled use of digital devices or digital content. Such excessive behavior can easily solidify into habits, thereby continuously affecting an individual’s emotions, cognition, and behavior (Cemiloglu et al., 2022). However, when the concept of addiction is directly applied to the elderly population, its applicability requires a dialectical analysis. For some older adults with limited social connections, declining mobility, or extended time spent at home, digital devices play an irreplaceable role in information access, emotional connection, and social participation. Prolonged use may be an adaptive or compensatory behavior rather than a pathological manifestation (Haase et al., 2025). However, when digital use gradually becomes uncontrolled and comes at the expense of physical health, real-life social relationships, and daily functioning, the characteristics of addiction become apparent. However, there is currently a lack of operational definitions and diagnostic criteria tailored to the unique physiological, psychological, and social roles of older adults. This forms a fundamental barrier to research progress in terms of conceptual clarification and measurements.

Compared to other age groups, the formation mechanism of digital addiction among the elderly is typically the result of interactions among multiple factors, such as digital technology stress, socio-cultural pressure, and individual emotional changes (Kim et al., 2023). For elderly individuals, certain physiological, psychological, and social vulnerabilities make them more susceptible to the effects of excessive use in a highly pervasive digital environment. For example, after retirement, an increase in free time and changes in social roles and daily routines can weaken their original time structure and behavioral constraints, making digital media an important tool for filling daily gaps (Xu L. R. et al., 2025). Furthermore, real-life challenges, such as chronic illness, physical decline, and limited mobility, may further reinforce their reliance on low-cost, easily accessible digital entertainment and online interactions (Wang et al., 2023). In addition, as traditional social networks shrink and real-world social opportunities decrease, the elderly’s need for emotional support and social connection does not diminish; on the contrary, it may be met through digital platforms as a substitute. Within this broader context, some studies have revealed specific mechanisms at the psychological, social, and individual cognitive levels. First, in terms of psychological vulnerability, loneliness is a key driver that encourages older adults to turn to the digital world for social compensation. When online interactions fail to provide the expected satisfaction, feelings of loneliness may intensify, thereby reinforcing dependence on digital devices (He et al., 2020). In addition, older adults with symptoms of depression or anxiety may immerse themselves in online activities to avoid negative emotions or real-life stress. This negative emotional regulation strategy increases the risk of addiction to social media. Second, at the level of social and environmental factors, a tense or unsupportive family atmosphere may prompt older adults to seek emotional comfort or temporarily escape real-life conflicts through Internet use (Reyes, 2025). On the other hand, emotional support and social care from family members, friends, and the community are regarded as important protective factors, helping to meet the elderly’s emotional and social needs and reducing excessive dependence on virtual interactions. Lastly, in terms of individual characteristics and cognitive factors, older adults who tend to adopt avoidant coping strategies are more likely to use the Internet as an escape, and high emotional instability or low conscientiousness is also associated with an increased risk of digital addiction (Zhang M. X., 2023). In summary, digital addiction among the elderly results from the interaction of macroenvironmental factors, psychological needs, and individual characteristics. Their usage behavior can gradually evolve from functional tool use to emotional regulation and psychological compensation, thereby increasing the risk of excessive digital immersion and even addiction.

However, the new generation of older adults is gradually becoming an important demographic in the use of digital technologies. Their digital usage experience, familiarity with technology, and social backgrounds differ from those of traditional older adults. This means that the driving forces, risk structures, and psychosocial impacts of digital addiction among the new generation of older adults may present new characteristics. In addition to motives such as compensating for loneliness and social disengagement, their excessive use of digital technology may also stem from the continuation of a long-term digital lifestyle, platform stickiness mechanisms, and persistent social pressure to stay online. However, current research lacks detailed distinctions among different elderly groups, making it necessary to further advance the study to more comprehensively and thoroughly reveal the unique mechanisms of digital addiction among the new generation of old adults and its social consequences.

### 2.3 Research gap

Existing research on digital participation among older adults largely continues to use broad and homogenous target groups, often treating older adults as a single unified cohort or making only crude distinctions based on age. This approach fails to reflect the emergence of new generation of older adults as a distinct intergenerational group in the context of digital transformation. Compared to traditional older adults, the ways in which the new generation of older adults integrates digitally, as well as the resulting risks, present new characteristics that require further research. However, most existing studies adopt a perspective centered on instrumental rationality or capability deficits, emphasizing technological accessibility and functional support while paying limited attention to the subjective agency, usage motivations, and complex experiences of older adults in digital practices. Consequently, they struggle to explain why active digital engagement may be accompanied by problematic use. Although some studies have recognized the phenomenon of digital addiction among older adults, their findings are mostly descriptive or exploratory, lacking systematic explanations and theoretical frameworks for its formation. Some research simply applies addiction frameworks developed for younger populations without sufficiently considering the unique contexts and psychological needs of the new generation of older adults as they transition into retirement and experience life-course changes. In particular, there is a lack of in-depth exploration of the mechanisms by which digital addiction gradually emerges during the process of active digital engagement, thus failing to address the core paradox of empowerment and risk coexistence within digital inclusion research. Furthermore, current research and practical strategies often separately address promoting digital inclusion and preventing digital addiction, lacking an integrated perspective and actionable framework. Digital inclusion serves not only instrumental needs but also fulfills the profound motivations of the new generation of older adults in their third age to reconstruct self-identity, seek meaning, and affirm their value. However, this perspective mostly remains at the theoretical or small-scale qualitative research level, lacking systematic empirical support. More importantly, almost no research has integrated the positive process of identity reconstruction with the risks of digital addiction, thereby missing a crucial entry point for revealing the internal mechanisms behind the paradoxes of digital inclusion and exclusion.

The possible innovations of this study are as follows. First, by focusing on the new generation of older adults as the main research subjects and introducing identity reconstruction into the core of the analysis of digital inclusion, this study reveals the internal mechanism behind the coexistence of active digital inclusion and the risk of digital addiction among the new generation of older adults. This study systematically clarifies the conditions under which digital empowerment is associated with digital addiction, thus providing a mechanistic theoretical path to explain the paradox of digital inclusion and expand the boundaries of theoretical analysis. Second, unlike existing studies that view identity as a static concept, this study divides identity reconstruction into five stages. It systematically depicts the dynamic evolutionary path of the new generation of older adults in digital inclusion, revealing that digital inclusion may generate empowering effects at certain stages while also accumulating addiction risks at others. This provides a clear theoretical basis for understanding the process-based mechanism underlying the generation of the digital inclusion paradox. Third, this study systematically examines the differentiated effects of identity reconstruction on digital addiction in different technological and cognitive contexts, introducing algorithmic perception, platform pressure, and digital reflection as key moderating variables, thereby revealing the contextual generation conditions of the digital inclusion paradox. Finally, based on the overall validation of the theoretical model’s applicability, this study further identifies multiple paths to the formation of digital addiction under various combinations of conditions, effectively responding to the paradoxical features of the coexistence of empowerment and risk, as well as the multiple and nonlinear mechanisms in digital inclusion research, thereby enhancing the explanatory power and applicability of the research conclusions for real-world digital governance and intervention practices.

## 3 Research framework and hypothesis

### 3.1 Theory framework

Digital inclusion is widely understood as a means of helping older adults bridge the digital divide and participate more fully in contemporary social lives. However, the expansion of digital inclusion has also brought the unintended consequence of digital addiction. This tension gives rise to the paradox of digital inclusion, whereby the same process that empowers older adults may also expose them to the risks of excessive and dependent use. This study seeks to explain how digital empowerment and the risk of digital addiction coexist among the new generation of older adults during digital inclusion. Departing from prior research that primarily attributes digital addiction to technological dependence or psychological compensation, we argue that digital inclusion among the new generation of older adults is fundamentally an identity reconstruction process embedded in life course transitions and shifting social roles. As they exit the workforce and lose access to traditional channels of participation and recognition, older adults face an urgent need to renegotiate their identities and how their social value is affirmed (Mai and Hao, 2020). Digital technologies and platform spaces provide a crucial arena for reconstruction. In this sense, digital inclusion is not merely a matter of access, skills, or usage; it is also a process through which the new generation of older adults rebuilds the self in digitally mediated contexts. This process helps explain why digital inclusion can generate both empowerment and addiction risk.

Dramaturgical theory provides an important perspective for understanding how these processes unfold. By conceptualizing social interaction as situated role performance, dramaturgical theory highlights how identities are constructed, negotiated, and sustained through impression management and interactive feedback (Whittle et al., 2021). In digital contexts, the new generation of older adults uses platforms as stages and interacts with others and algorithms as audiences. Through content production, self-presentation, and repeated interactions, they do not simply participate online; they actively perform and stabilize their digital identities. From this perspective, identity reconstruction can be conceptualized as a multistage process encompassing identity formulation, identification, dissemination, co-construction, and maintenance. These stages capture the movement from tentative self-positioning to increasingly stable digital roles. As identity dissemination and co-construction deepen, the digital stage becomes more than a tool for participation; it becomes a key site through which recognition, belonging, and self-worth are secured. Consequently, identity maintenance demands increasing investments of time, attention, and emotion, and digital engagement gradually shifts from instrumental use to emotional and symbolic attachment.

While dramaturgical theory explains how identity reconstruction unfolds, MOA theory clarifies why it is progressively reinforced. The MOA theory suggests that sustained behavior emerges through the interaction of motivation, opportunity, and ability (Chen and Cheng, 2026). In the case of the new generation of older adults, motivation arises from the need to reaffirm self-worth, reconstruct social roles, and sustain meaningful participation after major life transitions. Platform environments that facilitate visibility, interaction, and feedback provide opportunities. Ability develops through the accumulation of digital skills, familiarity with platform logic, and experience in managing online relationships. Together, these conditions transform identity reconstruction from a temporary adjustment to a durable and self-reinforcing practice. Digital inclusion thus moves beyond initial contact and functional use to become an identity-based routine that is deeply embedded in everyday life. Thus, the same process that supports adaptation also creates the behavioral conditions under which digital dependence may emerge.

Optimal distinctiveness theory further explains why identity reconstruction produces both empowering and problematic outcomes for individuals. The theory holds that individuals continuously seek to balance two competing yet complementary needs: belonging and uniqueness. For the new generation of older adults, digital inclusion satisfies the need for belonging by expanding social ties, increasing interaction frequency, and reducing the experience of marginalization. Simultaneously, it satisfies the need for distinctiveness by enabling content creation, identity labeling, and the accumulation of influence, thereby allowing older adults to express their individuality and affirm their personal value. In the early stages of identity reconstruction, this dual satisfaction underpins the positive effects of digital inclusion, including stronger participation, greater self-confidence, and enhanced, perceived social value. However, as identity dissemination, co-construction, and maintenance deepen, the satisfaction of belonging and distinctiveness becomes increasingly tied to persistent online presence, high-frequency interaction, and immediate feedback. Under these conditions, digital platforms cease to function merely as instruments of identity expression and instead become conditions for identity stability. At this point, empowerment may gradually give way to dependence, and digital inclusion may begin to generate the risk of digital addiction.

**Figure 1:**
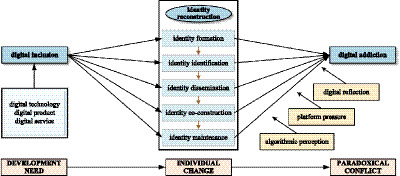
Theoretical framework of the digital inclusion paradox.

In summary, this study develops an integrated framework to explain the paradox of digital inclusion among the new generation of older adults. In this framework, dramaturgical theory explains the staged unfolding of identity reconstruction, MOA theory specifies the behavioral conditions that sustain and intensify it, and optimal distinctiveness theory explains why the same process can simultaneously produce empowerment and addiction risk. Digital inclusion is therefore conceptualized as a dynamic process of multistage identity reconstruction through which the new generation of older adults moves from initial adaptation and participation toward relatively stable forms of digital self-construction. Although this process enhances social connectedness and self-efficacy, it may also foster digital dependence when identity maintenance becomes increasingly reliant on platform-based interactions and feedback. To capture variations across digital contexts, the framework incorporates algorithmic perception, platform pressure, and digital reflection as key boundary conditions shaping the strength of the relationship between identity reconstruction and digital addiction. Based on the theoretical integration above, this study ultimately establishes a theoretical framework (see Figure 1), which offers a stronger theoretical basis for understanding the paradox of digital inclusion and promoting healthier and more sustainable forms of digital inclusion among the new generation of older adults.

### 3.2 Research hypothesis

#### 3.2.1 Main path and mediating effect

As the first transitional generation to enter the digital society, the new generation of older adults tends to deeply integrate digital technology into their daily lives, social interactions, and self-expression, forming an ongoing, proactive, and contextualized process of digital inclusion. However, the impact of this process goes beyond increased usage frequency or technical proficiency; its deeper significance lies in reshaping self-identity and social roles. Specifically, the identity reconstruction of the new generation of older adults exhibits notable diversity, further confirming the central role of identity transformation in their digital behavior (Altanlar et al., 2025). On social and lifestyle platforms, the new generation of older adults proactively curate, edit, and consistently share content, showcasing youthful lifestyles such as travel, fitness, learning, and aesthetic taste. Through this, they construct a positive, active digital self-image that resists being labeled by age, thereby countering the self-threat brought about by physical aging and the age discrimination present in society. However, leveraging their long-accumulated professional knowledge and life experience, some new generations of older adults are gradually emerging as silver-haired opinion leaders in specific fields. Through knowledge sharing, passing on experience, and emotional engagement, they reaffirm their self-worth and reshape the modern social image of wise old people. Furthermore, various virtual communities formed around shared interests, hobbies, and values provide the new generation of older adults with new spaces for social belonging that extend beyond family roles and retirement identities. In these decentralized, low-hierarchy interaction settings, they participate more as members or partners rather than in traditional intergenerational roles, thereby rebuilding social capital and, to some extent, filling the emotional and identity gaps caused by the weakening of real-world social networks after retirement.

It is precisely through ongoing interaction, repeated affirmation, and emotional investment in the aforementioned digital practices that identity reconstruction gradually evolves from an exploratory self-attempt into a behavioral pattern characterized by stable expectations and path dependence. As digital platforms continuously amplify instant feedback, social recognition, and emotional rewards brought about by identity expression, the psychological attachment and behavioral stickiness of the new generation of older adults to digital contexts likewise increase. Thus, while digital inclusion has empowering effects, it also harbors the potential risk of evolving into digital addiction. On this basis, this study contends that digital inclusion does not directly influence digital addiction among the new generation of older adults; its key mechanism of influence lies in driving the systematic reconstruction of their digital identity. Identity reconstruction is a multilevel process that can be divided into five interrelated core dimensions. The following section presents a systematic analysis of these dimensions.

##### 3.2.1.1 Digital inclusion → identity formation → digital addiction

In the process of deepening digital inclusion, the new generation of older adults no longer sees digital technology merely as a tool for completing specific tasks but gradually transforms it into an important arena for self-definition and self-presentation (Rasi-Heikkinen and Doh, 2023). Compared to traditional elderly groups, the new generation of older adults exhibits a stronger sense of agency and a tendency toward self-planning. When joining social, content, or lifestyle platforms, users actively consider how they wish to be perceived by others. This repeated selection and adjustment of personal profiles and content themes form the core process of digital identity construction. As the level of digital inclusion increases, identity-forming behaviors become more frequent and refined, causing a new generation of older adults to psychologically view digital spaces as a crucial extension of themselves. When they continually invest cognitive and emotional resources to maintain the digital identities they have established, their dependence on these platforms increases, thereby raising the possibility of a digital use imbalance or even addiction (Taipale et al., 2021). Therefore, the following hypothesis is proposed: the higher the level of digital inclusion, the more likely the new generation of older adults is to actively construct and shape their digital identities in digital spaces. This ongoing investment in identity construction increases their psychological dependence on digital media, thus elevating the level of digital addiction.

##### 3.2.1.2 Digital inclusion → identity identification → digital addiction

Once a digital identity is used continuously and receives positive feedback, the new generation of older adults no longer remains at the surface level of image construction; instead, it gradually becomes an important source for their self-concept. Retirement and changes in social roles often weaken the channels through which they can affirm their identities in real-life situations. In contrast, the instant feedback, emotional support, and social visibility provided by digital spaces are crucial supplements for regaining a sense of being needed and recognized (Choudrie et al., 2020). As digital inclusion deepens, the new generation of older adults is more likely to develop emotional attachment to and psychological identification with the identity they form on digital platforms, regarding it as an extension of their real self. This high level of identification not only enhances their sense of presence in digital environments but also makes them increasingly reliant on digital contexts for emotional regulation and self-affirmation, thereby increasing the risk of continuous and excessive use (Siren and Knudsen, 2017). Therefore, the following hypothesis is proposed: digital inclusion indirectly promotes digital addiction among the new generation of older adults by enhancing their sense of identification with their digital identity.

##### 3.2.1.3 Digital inclusion → identity dissemination → digital addiction

Under the influence of algorithmic mechanisms and social logic on digital platforms, identity is not static but requires continual dissemination and display to receive stable social responses. After the new generation of older adults completes the initial process of identity construction and forms an identity recognition, they reinforce the social visibility of their digital identity by continuously publishing content, engaging in interactions, and showcasing their lifestyles (Chen and Hartt, 2021). This behavior of identity dissemination is particularly evident among digitally integrated members of the new generation of older adults; it is not only an act of information sharing but also a way to maintain social connections and affirm self-worth. However, identity dissemination relies heavily on feedback mechanisms provided by platforms, such as likes, comments, and follows. This external reinforcement easily leads to prolonged online time and increased usage frequency (Yang and Du, 2021). When identity dissemination becomes an important channel for obtaining emotional satisfaction and social recognition, the attachment of the new generation of older adults to digital platforms is significantly enhanced, thereby increasing their tendency toward digital addiction. Therefore, the following hypothesis is proposed: the higher the level of digital inclusion, the more frequently new generation older adults will engage in overt expression and dissemination of their digital identities. The social feedback and reinforced visibility brought about by identity dissemination further deepens their dependence on digital platforms, thus increasing their level of digital addiction.

##### 3.2.1.4 Digital inclusion → identity co-construction → digital addiction

Digital identity is not shaped unilaterally by individuals alone; rather, it is continuously confirmed, revised, and co-constructed by others through ongoing interactions. For the new generation of older adults, online communities formed around shared interests, values, or life experiences provide a relatively equal and low-age-stereotyped environment for interaction. In these contexts, the identity of new generation of older adults is often reinforced through others’ responses, expectations, and role assignments, forming a typical process of mutual identity construction (Mikolajczyk, 2023). As digital inclusion deepens, older adults participate in such interactions more frequently and assume relatively stable interactive roles within their social networks. The sense of social embeddedness and emotional connection brought about by mutual identity construction makes them increasingly psychologically reliant on digital communities as important sources of social support, thereby, often unconsciously, enhancing their motivation to stay online and their behavioral inertia, raising the risk of digital addiction (Hänninen and Tiihonen, 2026). Therefore, the following hypothesis is proposed: digital inclusion, by promoting ongoing mutual digital identity construction with others during digital interactions among older adults of the new generation, enhances their sense of presence and relational embeddedness in interactive contexts, thereby increasing their level of digital addiction.

##### 3.2.1.5 Digital inclusion → identity maintenance → digital addiction

After their digital identity stabilizes through repeated interactions and feedback, the new generation of older adults often invests more resources to maintain their existing identity status. This identity maintenance not only means continually updating content or keeping up the frequency of interactions, but also includes the psychological motivation to avoid identity weakening, social distancing, or a decline in social visibility (Cunningham et al., 2025). As the degree of digital inclusion increases, the new generation of older adults becomes more concerned with the stability of their identity, which in turn creates constant pressure to stay online. This identity-driven digital usage tends to exhibit strong inertia, leading them to repeatedly access digital platforms even in the absence of clear usage goals, thereby increasing the risk of uncontrolled digital use (Zhang Y. Q., 2023). Therefore, the following hypothesis is proposed: digital inclusion, by strengthening the ongoing maintenance of their existing digital identity among the new generation of older adults, enhances their path dependency on digital spaces, thereby affecting their digital addiction.

#### 3.2.2 Moderating effect

##### 3.2.2.1 The moderating effect of algorithm perception on identity reconstruction and digital addiction

In the highly algorithm-driven environment of digital platforms, the subjective perception of algorithms by the new generation of older adults profoundly influences the psychological outcomes of their digital behavior. Once older adults reconstruct their identities in digital spaces, the identity images, content styles, and interaction patterns they exhibit are more easily captured and amplified by algorithms (Zhang and Liu, 2025). In this process, if the new generation of older adults lacks a clear understanding of how algorithms function or tends to equate algorithmic feedback with genuine social recognition, the positive reinforcement resulting from their identity reconstruction is more likely to be magnified into highly emotional, intensified experiences. In particular, in situations where channels for social identity confirmation in real life are relatively limited, the new generation of older adults is more inclined to view algorithmic promotion and exposure as objective proof of their self-worth, thereby deepening their psychological attachment to digital contexts and making identity reconstruction more likely to translate into ongoing, high-frequency digital behaviors (Chen et al., 2024). Conversely, if the new generation of older adults can clearly recognize the selectivity and instrumental nature of algorithms, their emotional responses to feedback will be relatively restrained, thereby weakening the intensity with which identity reconstruction translates into digital addiction. Based on this, the following hypothesis is proposed: algorithm perception moderates the relationship between identity reconstruction and digital addiction in a negative direction; that is, when the new generation of older adults have a higher level of perception of platform algorithms, the impact of identity reconstruction on digital addiction is weaker.

##### 3.2.2.2 The moderating effect of platform pressure on identity reconstruction and digital addiction

Apart from algorithmic mechanisms, platform codes of conduct and implicit expectations also constitute important contextual factors that influence the outcomes of identity reconstruction processes. Platform pressure mainly manifests as implicit expectations regarding content update frequency, interaction response speed, and maintaining a continuous online presence. This pressure does not exist in the form of mandatory rules but is continually reinforced through platform design logic and community interaction norms (Ochinanwata et al., 2024). After the new generation of older adults complete their identity reconstruction and establish stable interactive relationships, they begin to feel sustained expectations from both the platform and other users, such as the inability to stop updating for long periods or the need to respond to interactions promptly. To a certain extent, this pressure strengthens the sense of responsibility to maintain and perform their identity, gradually transforming the digital identity from a tool for self-expression into a role burden that requires ongoing management (Xu J. et al., 2025). When platform pressure is high, the new generation of older adults is more likely to extend their online time out of concern for weakened identity, loss of relationships, or reduced social visibility, thereby intensifying the shift from identity reconstruction to digital addiction. In contrast, in situations where platform pressure is low, the new generation of older adults has greater autonomy in their digital identity involvement, and their usage behaviors are more likely to remain flexible. Based on this, the following hypothesis is proposed: Platform pressure positively moderates the relationship between identity reconstruction and digital addiction; that is, when perceived platform pressure is high, identity reconstruction among the new generation of older adults is more likely to trigger digital addiction.

##### 3.2.2.3 The moderating effect of digital reflection on identity reconstruction and digital addiction

Although identity reconstruction may increase the risk of digital addiction, it is not irreversible and can be undone. The key lies in whether the new generation of older adults possesses the ability to reflect on and regulate their digital behavior. Digital reflection refers to the tendency of the new generation of older adults to actively examine and evaluate their motivations for digital use, the time they invest in it, and its consequences. Compared to younger groups, the new generation of older adults generally has richer life experiences and a more stable value system, enabling them to remain more alert to the boundaries between their digital identity and their real self when they possess reflective awareness (Wang and Wu, 2022). When digital reflection is at a high level, the new generation of older adults is more likely to recognize that the emotional satisfaction and social feedback resulting from identity reconstruction are contextual and temporary. As a result, they consciously adjust their usage patterns to avoid becoming overly immersed in their digital roles (Ma et al., 2026). In this context, even if the degree of identity reconstruction is high, its effect on driving digital addiction is weakened. Conversely, if the level of digital reflection is low, the new generation of older adults is more likely to unconsciously continue along the path of identity reinforcement and the pursuit of feedback, thereby amplifying the risk of addiction. Based on this, the following hypothesis is proposed: digital reflection negatively moderates the relationship between identity reconstruction and digital addiction; that is, when the level of digital reflection among the new generation of older adults is high, the impact of identity reconstruction on digital addiction is weaker.

## 4 Research design

### 4.1 Empirical methodology

Given the paradoxical characteristics observed in the digital inclusion process of the new generation of older adults, namely, that while digital inclusion promotes identity reconstruction and social participation, it may also have consequences such as digital addiction, this study focuses on a complex causal system encompassing potential psychological mechanisms, mediating pathways, and multiple conditional interactions. To systematically reveal the core mechanism of “digital inclusion-identity reconstruction-digital addiction”, both SEM and fsQCA are employed, using complementary perspectives of linear causal effect testing and configurational causal mechanism identification. SEM, as a multivariate analysis method capable of simultaneously handling latent variable measurement and structural relationship estimation, is well-suited for testing theoretical models involving abstract concepts and impact effects (Liu et al., 2025). In the context of this study, the key variables, digital inclusion, identity reconstruction, and digital addiction, all have multidimensional and latent characteristics. SEM can effectively reduce measurement error by incorporating multiple observed indicators, accurately estimate the direct effect of digital inclusion on digital addiction within an overall model framework, assess the mediating role of identity reconstruction, and identify the net effect and strength of each pathway by controlling for individual characteristic variables. This provides rigorous statistical support for the linear causal test of the paradoxical mechanism in this study. However, SEM mainly explains average effects and is limited in fully revealing the diverse causal pathways that may arise from different combinations of conditions in specific contexts. To address this limitation, fsQCA is further introduced to portray, from a set-theoretic and configurational perspective, the combined impact of various antecedent conditions on the outcome variable under different configurational states (Rasoolimanesh, 2026). This method helps identify multiple pathways forming digital addiction in the new generation of older adults based on combinations of digital inclusion and identity reconstruction status. The complementary application of these two methods not only enhances the robustness and explanatory power of the research conclusions but also provides a more comprehensive analytical perspective for fully understanding the paradoxical mechanism of digital inclusion among the new generation of older adults.

### 4.2 Questionnaire design

Given the diversity of digital inclusion forms and the complexity of the measurement, this study focuses on the digital inclusion of the new generation of older adults on short video platforms. On the one hand, short videos have already become highly popular and frequently used among the elderly, serving as the most representative application in their digital lives. On the other hand, by continuously enhancing content interaction, social connection, and identity expression features, short video platforms have enabled the new generation of older adults to participate in ways that go beyond mere information reception, embedding these platforms deeply into their daily routines and self-understanding. Therefore, using short video platforms as the specific research context for digital inclusion helps improve the contextual focus and theoretical explanatory power of the questionnaire and offers more practical guidance for examining the relationships among digital inclusion, identity reconstruction, and digital addiction among the new generation of older adults.

Digital inclusion is characterized by strong abstraction and multidimensionality. In the questionnaire design process, both the overall structure and item formulation focused on balancing theoretical rigor with practical measurability while emphasizing a clear focus on the research context. In terms of design principles, the questionnaire followed a progression from simple to complex and adopted a step-by-step organizational logic. The wording of the items was tailored as closely as possible to the everyday short video usage experiences and cognitive levels of the new generation of older adults, reducing abstract and specialized expressions to enhance respondents’ understanding and the consistency of their answers. During the specific design process, mature scales that have demonstrated good reliability and validity in relevant research fields, such as digital inclusion and digital addiction, were consulted (Andreassen et al., 2012; Leung, 2008), and items were selected and refined to ensure consistency with theoretical concepts. Additionally, based on the theoretical path of “digital inclusion-identity reconstruction-digital addiction”, the core variables were conceptually defined and broken down into observable measurement dimensions, forming the preliminary item system. The main focus is on the possible occurrences of time loss of control and habitual usage during sustained digital inclusion among the new generation of older adults, while also retaining their underlying positive motivations, such as learning new knowledge, maintaining social connections, and continuous participation in public life. This approach reframes digital addiction not as a negative outcome in opposition to digital inclusion but as a paradoxical consequence that may arise alongside the pursuit of value, thereby aligning logically with the mediating mechanism of identity reconstruction. Furthermore, considering the usage characteristics of short video platforms, such as low barriers to participation, frequent content interaction, and prominent social display, some items were contextually adjusted to strengthen the alignment between the scale and the digital practices of the study population. During the questionnaire finalization phase, researchers from relevant fields were invited to review the theoretical alignment, clarity of expression, and potential ambiguities of the items, with several rounds of revision made in response to feedback. Prior to official distribution, a small-scale pilot test was conducted to examine the difficulty of comprehension, response consistency, and overall completion time, and the questionnaire structure and wording were refined accordingly. The final official questionnaire consisted of three parts: questionnaire instructions, basic information about respondents, and the core measurement scale (Table 1).

**Table 1:** Questionnaire items.

Number	Item content
DI1	I learn useful knowledge for myself, such as health and wellness and life tips, from short videos.
DI2	I keep up with current events through short videos, which makes me feel in touch with the times and able to converse with young people.
DI3	I use group buying and shopping features in short videos to make my life more convenient.
IF1	I will share my professional knowledge or life experiences through short videos.
IF2	I enjoy recording and sharing my hobbies using short videos.
IF3	I share my views on social events or trending topics through comments or by posting videos on social media.
IR1	When my sharing is helpful to others, I feel fulfilled.
IR2	In short video communities, I still feel like I can provide value to others.
IR3	Friendly interactions with young people or other Internet users make me feel like I am not outdated.
ID1	I hope my experiences can be shared with more young people through short videos to help them avoid unnecessary detours.
ID2	I enjoy communicating with young people through short videos, taking on the role of someone who has experienced everything.
ID3	I hope that by sharing my experiences, I can spread a positive and rational attitude toward life.
IC1	I have learned a lot of new things and new perspectives by reading other people’s comments and watching videos.
IC2	Discussions with online friends sometimes change the way I see myself or certain things.
IC3	I believe that my image on short videos is something that has been shaped by me and everyone through our interactions.
IM1	To continuously share valuable content, I make an effort to learn new knowledge and skills.
IM2	Even if I have difficulty filming or editing videos, I will do my best to overcome it because I believe it is meaningful.
IM3	Continuously updating short videos is mainly for self-improvement and staying energized, not to please the fans.
AP1	I know that short video apps decide what to show me next based on the content I like and watch.
AP2	I feel like the short videos I am seeing are becoming increasingly similar, as if I am trapped in a bubble.
AP3	I understand that the content recommended to me by short video platforms can influence my views on certain people and events.
PP1	Seeing the great videos made by other people my age makes me feel anxious, as if I am not doing well enough.
PP2	I am worried that my knowledge and experience are insufficient and that I will not be able to continuously share valuable content.
PP3	There are so many comments and private messages that I need to respond to, and sometimes, it can feel overwhelming.
DR1	I will assess whether watching short videos helps me gain more knowledge or just wastes too much of my time.
DR2	When I realize that watching videos has deviated from my original intentions (such as learning or socializing), I proactively make adjustments.
DR3	I will consciously choose to focus on information that is truly useful to me and ignore irrelevant content.
DA1	I originally just wanted to learn something or check in on friends, but I often end up scrolling for a long time without realizing it.
DA2	Even if there is nothing I want to watch, I habitually open short video apps.
DA3	If I go a day without watching short videos, I feel like I have missed out on a lot of important information and cannot quite put my mind at ease.

### 4.3 Sample selection and data collection

This study targets the new generation of older adults, focusing on those with relatively sound cognitive and physical functions who are capable of actively engaging in digital interactions, making them more likely to experience identity reconstruction through digital media. To ensure that the sample closely aligned with the research topic, the study limited its participants to elderly people aged between 61 and 76 years who had used short video platforms at least three times per week in the past six months. To enhance sample representativeness and structural diversity, a multistage sampling strategy was adopted. First, purposive sampling was used to select cities with typical characteristics in terms of digital economy development and the degree of population aging as survey areas. Next, within these selected cities, a combination of online and offline convenience sampling was employed to cover new-generation elderly groups with differing levels of digital literacy. Online recruitment was primarily conducted by posting survey invitations on social media platforms and senior interest communities, supplemented by snowballing, whereby respondents were encouraged to forward the survey link to peers of similar age and short video usage backgrounds. Offline recruitment was carried out through cooperation with senior universities, community activity centers, and elderly care service institutions, where in-person invitations and organized questionnaire completions were conducted.

Data collection was primarily conducted through the distribution and retrieval of questionnaires on a professional online survey platform. For respondents recruited offline, researchers provided tablet devices on-site and offered the necessary operational guidance to minimize systematic bias caused by technological barriers. Rigorous data quality control and screening procedures were implemented after the questionnaires were collected to ensure the authenticity and validity of the data. Specifically, questionnaires meeting any of the following conditions were considered invalid and excluded: (1) anomalous total response time, that is, significantly less than 30% of the pretest average completion time or more than three times that duration; (2) failure to correctly answer 2–3 attention check questions embedded in the survey; (3) highly consistent, straight-line, or repetitive response patterns in scale items; (4) obvious contradictions in answers to logically related questions; (5) failure to meet screening criteria set at the beginning of the questionnaire, such as age and frequency of platform use. A total of 1,190 questionnaires were distributed in this study, with 800 valid questionnaires ultimately obtained, serving as the sample base for subsequent statistical analysis. The basic information of the survey participants is presented in Table 2. The sample mainly consisted of the younger segment of the new generation of older adults, who generally had relatively good physical and cognitive abilities and were capable of ongoing participation in digital activities. This helps provide a comprehensive reflection of the actual digital inclusion of the new generation of older adults and serves as a fundamental guarantee of the validity and reliability of the data. In terms of socioeconomic characteristics, most respondents had work experience in enterprises and mainly resided in Tier-1, new Tier-1, and Tier-2 cities, indicating that the sample as a whole had a relatively high level of urbanization and a stable basis for social participation. Their living environments and public service conditions create a favorable external context for exposure to and use of digital technology. Regarding digital abilities and usage behavior, most respondents have already mastered the operation of mobile phones and computers fairly proficiently, and the multistage of digital platforms is generally lengthy, demonstrating that the new generation of the elderly has, to a certain extent, already integrated into the digital life.

**Table 2:** Basic information statistics of survey participants.

Statistical item	Option	Frequency
Age	61–65 years old	454
	66–70 years old	267
	71–76 years old	79
Occupation	Government agency/public institution	33
	Enterprise	706
	Freelancer/self-employed individual	61
Residence	Tier-1 cities	179
	New tier-1cities	270
	Tier-2 cities	242
	Tier-3 and lower-tier cities	109
Proficiency with mobile phones and computers	Highly proficient	215
	Fairly proficient	415
	Basic proficient	170
Daily digital platform usage time	1–2 h	328
	3–5 h	383
	5 h or more	89

## 5 Empirical result and discussion

### 5.1 Preliminary inspection

#### 5.1.1 Reliability and validity analysis

To assess the consistency of the measurement results, a systematic reliability test was conducted on all valid samples. The results (Table 3) show that both at the overall level and across each latent dimension, the data exhibited a high level of reliability. The Cronbach’s alpha for the overall data was 0.880, which meets the commonly used criteria in existing research. Simultaneously, the Cronbach’s alpha values for each dimension fell within the standard range, indicating strong consistency and stability among the measurement items. Therefore, the data collected in this study demonstrate robust reliability, providing a solid foundation for subsequent model testing and empirical analyses.

**Table 3:** Reliability test.

Variable	Number of measurement items	Cronbach’s alpha	Reliability
DI	3	0.844	High
IF	3	0.811	High
IR	3	0.842	High
ID	3	0.814	High
IC	3	0.831	High
IM	3	0.868	High
AP	3	0.841	High
PP	3	0.881	High
DR	3	0.847	High
DA	3	0.825	High
Totality	30	0.880	High

DI, digital inclusion; IF, identity formation; IR, identity identification; ID, identity dissemination; IC, identity co-construction; IM, identity maintenance; IY, identity reconstruction; AP, algorithmic perception; PP, platform pressure; DR, digital reflection; and DA, digital addiction.

In terms of validity testing, the KMO test and Bartlett test of sphericity were employed to assess the structural suitability of the data. As shown in Table 4, the KMO test value is 0.903, which is considered high, indicating that the sample data demonstrate a strong factor aggregation characteristic. At the same time, the result of Bartlett’s test of sphericity was less than 0.05, which is highly significant statistically. Taken together, these results suggest that the sample data exhibit good structural validity, effectively capturing the latent characteristics and cognitive structure of the research subjects, thus ensuring the scientific rigor and credibility of the subsequent empirical analysis.

**Table 4:** Validity test.

Test		Result
Kaiser-Meyer-Olkin measurement of sampling adequacy		0.903
Bartlett test of Sphericity	Approximate chi-square	12480.619
	Degrees of freedom	435
	Statistical significance	0.000

#### 5.1.2 Goodness-of-fit test

To evaluate the rationality and robustness of the theoretical model, a systematic assessment of the model fit was conducted. When multiple fit metrics simultaneously reach standards widely accepted in academia, it can be considered that the model exhibits a high degree of consistency with the observed data. The overall model fit was thoroughly examined from three perspectives: absolute fit, incremental fit, and parsimony fit. As shown in Table 5, all metrics met the recommended thresholds, indicating that the model demonstrated a good fit across various evaluation dimensions. In particular, even when both model complexity and explanatory power are considered, the relevant parsimony indices remain within a reasonable range, suggesting that the model has not introduced excessive parameters while maintaining explanatory strength. Overall, the theoretical model constructed in this study accurately captured the structural relationships among the variables and exhibited a high degree of compatibility with the sample data. These results not only confirm the rationality of the model structure but also provide a reliable foundation for subsequent hypothesis testing.

**Table 5:** Fit index.

Metrics	Indicator	Adaptation criteria	Value	Conclusion
Absolute fit metrics	CMIN/DF	1 < CMIN/DF < 3	2.412	Ideal
	RMR	RMR<0.05	0.019	Ideal
	GFI	GFI>0.90	0.918	Ideal
	AGFI	AGFI>0.90	0.913	Ideal
	RMSEA	RMSEA<0.08	0.055	Ideal
Incremental fit metrics	NFI	NFI>0.90	0.927	Ideal
	IFI	IFI>0.90	0.947	Ideal
	TLI	TLI>0.90	0.937	Ideal
	CFI	CFI>0.90	0.947	Ideal
Parsimonious fit metrics	PGFI	PGFI>0.50	0.707	Ideal
	PNFI	PNFI>0.50	0.786	Ideal
	PCFI	PCFI>0.50	0.803	Ideal

### 5.2 Model evaluation

#### 5.2.1 Main influencing path analysis

Based on the theoretical model, this study empirically examined the paradoxical mechanism of digital inclusion among the new generation of older adults, focusing on the main causal pathways and their underlying logic. As shown in Table 6, digital inclusion exerts a significant positive effect on identity formation, identification, dissemination, co-construction and maintenance. Furthermore, it significantly promotes digital addiction. The direction and significance of the main pathways in the model aligned closely with the research hypotheses, confirming the robustness of the theoretical assumptions. These results not only demonstrate the strong explanatory power and fit of the constructed model in elucidating how digital inclusion influences digital addiction through identity reconstruction mechanisms but also reveal the more complex social motivations underpinning the digital behaviors of the new generation of older adults. Specifically, digital inclusion does not simply push the new generation of older adults into a passive state of technological dependence or media addiction; rather, it activates their identity construction processes, allowing them to continuously engage in self-definition, value affirmation, and the reproduction of social relationships on digital platforms. In this process, the new generation of older adults shifts from being passive recipients in the traditional sense to active constructors. They use digital technology to express their experiences, demonstrate their abilities, expand their social connections, and reinforce their identification and maintenance of personal roles through ongoing interactions and feedback. It is precisely this highly active practice of identity that embeds digital usage into their everyday lives and systems of meaning, thereby presenting the paradoxical feature of positive inclusion coexisting with the risk of addiction. This finding resonates deeply with the group profile of the new generation of older adults, who are concerned about how to live, emphasizing agency and self-worth. This suggests that digital addiction does not necessarily equate to functional decline or loss of control but may be a structural by-product of ongoing social participation and the pursuit of self-fulfillment, thus offering a more explanatory theoretical perspective for reinterpreting the digital behaviors of the new generation of older adults.

**Table 6:** Model path analysis and hypothesis verification.

Path relationship	Standardized path coefficient	Unstandardized path coefficient	Correlation	*P*	Conclusion
Identity formation ← Digital inclusion	0.539	0.503	Positive	***	Assumption established
Identity identification ← Digital inclusion	0.484	0.447	Positive	***	Assumption established
Identity dissemination ← Digital inclusion	0.556	0.566	Positive	***	Assumption established
Identity co-construction ← Digital inclusion	0.559	0.540	Positive	***	Assumption established
Identity maintenance ← Digital inclusion	0.500	0.540	Positive	***	Assumption established
Digital addiction ← Digital inclusion	0.063	0.054	Positive	***	Assumption established
Digital addiction ← Identity formation	0.192	0.177	Positive	***	Assumption established
Digital addiction ← Identity identification	0.140	0.130	Positive	***	Assumption established
Digital addiction ← Identity dissemination	0.166	0.140	Positive	***	Assumption established
Digital addiction ← Identity co-construction	0.140	0.125	Positive	***	Assumption established
Digital addiction ← Identity maintenance	0.138	0.110	Positive	***	Assumption established

P represents significance, and *** indicates significance at the 0.001 level.

#### 5.2.2 Mediating effect analysis

The Bootstrapping resampling method was employed to test the mediating roles of identity formation, identification, dissemination, co-construction, and maintenance between digital inclusion and digital addiction. Bootstrap resampling was set at 1000 iterations, and both bias-corrected and percentile confidence intervals were reported at a 95% confidence level to ensure the stability and reliability of the indirect effect estimates. As shown in Table 7, none of the confidence intervals for the five mediating paths (both Bias-Corrected and Percentile) included zero, indicating the presence of indirect effects. Therefore, identity formation, identification, dissemination, co-construction, and maintenance significantly mediated the relationship between digital inclusion and digital addiction, confirming the relevant hypotheses. Mechanistically, digital inclusion first stimulates the expression needs of the new generation of older adults, which are then met through interactive feedback that satisfies their need for contribution and gradually elevates their needs for influence. Through continuous interaction, the new generation of older adults establishes a reciprocal learning relationship with others, fulfilling both learning needs and promoting the deepening of identity co-construction. Ultimately, this series of positive feedback loops accumulates in an intrinsic pursuit of self-transcendence, manifesting as the maintenance of identity value through continuous learning and self-improvement. It is precisely in this process of identity construction, progressing from lower to higher levels step by step, that digital inclusion evolves from a utilitarian behavior into a meaningful practice, becoming deeply embedded in individuals’ value realization and the structure of daily life, thereby making digital addiction a process-driven outcome driven by needs and continually reinforced by identity reconstruction. This finding reveals that digital addiction among the new generation of older adults does not primarily originate from self-control failure or cognitive vulnerability but is rooted in their ongoing fulfillment of multilayered social and psychological needs and active participation in the practice of identity reconstruction. This provides a more explanatory basis for understanding the paradoxical features of digital behavior among the new generation of older adults.

**Table 7:** Mediation effect test.

Relationships	Point estimate	Product of coefficients		Bootstrapping					
				Bias-corrected 95% CI			Percentile 95% CI		
		S.E.	Z	Lower	Upper	*P*	Lower	Upper	*P*
Indirect effects									
DI → IF→DA	0.089	0.023	3.870	0.054	0.144	0.000	0.049	0.137	0.001
DI → IR → DA	0.058	0.021	2.762	0.027	0.111	0.000	0.024	0.104	0.001
DI → ID→DA	0.079	0.022	3.591	0.045	0.132	0.001	0.043	0.130	0.001
DI → IC → DA	0.067	0.023	2.913	0.030	0.123	0.001	0.028	0.118	0.001
DI → IM → DA	0.059	0.020	2.950	0.024	0.104	0.001	0.022	0.101	0.001
Total IE	0.353	0.051	6.922	0.259	0.457	0.001	0.256	0.456	0.001
Contrasts									
IF-IR	0.031	0.030	1.033	−0.028	0.093	0.282	−0.029	0.091	0.303
IR-ID	−0.021	0.030	−0.700	−0.077	0.039	0.498	−0.081	0.036	0.432
ID-IC	0.012	0.030	0.400	−0.047	0.071	0.685	−0.047	0.072	0.665
IC-IM	0.008	0.032	0.250	−0.052	0.074	0.771	−0.052	0.074	0.788
IM-IF	−0.030	0.030	−1.000	−0.092	0.026	0.277	−0.091	0.028	0.301

#### 5.2.3 Moderating effect analysis

To reveal the key boundary conditions of digital addiction among the new generation of older adults, further tests were conducted on the moderating effects of algorithmic perception, platform pressure, and digital reflection on the relationship between identity reconstruction and digital addiction in this group.

As shown in Table 8, algorithmic perception and digital reflection had significant negative moderating effects, whereas platform pressure had a significant positive moderating effect. Specifically, a higher level of algorithmic perception significantly weakened the promoting effect of identity reconstruction on digital addiction. This suggests that an individual’s understanding of the operational logic behind platform algorithms does not deepen their immersion; instead, it helps them comprehend the mechanisms of content distribution and the interactive feedback. This, in turn, diminishes the emotional reinforcement and behavioral dependence caused by algorithmic feedback, promoting a more rational and bounded approach to identity practices. Similarly, the negative moderating effect of digital reflection further indicates that older adults in the new generation with stronger reflective abilities can, while actively participating in identity construction, regulate their own digital behavior and avoid over-reliance on platform feedback for identity value, thus inhibiting the transition from identity reconstruction to digital addiction. In contrast, the significant positive moderating effect of platform pressure means that when new-generation older adults perceive external pressures from platform update rhythms, interaction metrics, exposure mechanisms, and regulatory requirements, identity reconstruction is more easily swept into performance-oriented and competitive dynamics. What originally began as identity practice driven by internal needs gradually turned into continuous responses to platform rules, thereby increasing reliance on digital media and accelerating the formation of digital addiction. In summary, whether identity reconstruction evolves into digital addiction largely depends on whether individuals possess sufficient algorithmic awareness and digital reflection abilities, as well as on the presence of ongoing pressure within their platform environment. These findings not only deepen the understanding of the mechanisms behind digital addiction among the new generation of older adults but also provide important empirical evidence and intervention directions for achieving healthy and orderly digital inclusion by enhancing algorithmic perception, strengthening digital reflection, and reducing platform use pressure.

**Table 8:** Moderation effect test.

Path relationship	Standardized path coefficient	Unstandardized path coefficient	C.R.	Correlation	*P*
DA ← IY × AP	−0.326	−0.199	−4.902	Negative	***
DA ← IY × PP	0.118	0.114	2.301	Positive	***
DA ← IY × DR	−0.497	−0.319	−7.181	Negative	***

### 5.3 Fuzzy-set qualitative comparative analysis

#### 5.3.1 Variable selection and data calibration

Digital inclusion, identity formation, identity identification, identity dissemination, identity co-construction, identity maintenance, algorithmic perception, platform pressure, and digital reflection were selected as explanatory condition variables, with digital addiction set as the outcome variable, to construct a multi-condition combination analytical framework. The selection of these variables aimed to systematically reveal the interactive effects and configuration outcomes of different organizational characteristics and situational elements in the formation of digital addiction. To improve the accuracy of the variable measurements and the comparability of the samples, the original data were directly calibrated. Specifically, drawing on the distribution characteristics of the sample data, 95, 50, and 5% were used as the key calibration anchors (Veríssimo, 2018), corresponding to complete membership, intersection, and complete non-membership points, respectively. Through this calibration process, continuous variables were converted into fuzzy set membership scores ranging from 0 to 1, effectively reflecting the relative position of the samples within different condition sets. The calibrated variables (Table 9) not only retain the differentiation information of the original data but also provide a clear and standardized quantitative foundation for subsequent necessary condition analysis and sufficient condition configuration analysis.

**Table 9:** Variable calibration.

Variable	Complete membership point	Intersection point	Complete non-membership point
DI	5.9	5.3	1.7
IF	6.2	5.4	1.9
IR	6.2	5.3	1.9
ID	6.0	5.3	1.9
IC	6.2	5.4	2.0
IM	6.2	5.3	1.9
AP	6.3	5.3	1.9
PP	5.9	2.5	1.5
DR	6.3	5.3	2.0
DA	6.2	5.3	2.0

#### 5.3.2 Necessary condition analysis and truth table construction

Before conducting the configuration analysis, we tested whether each individual condition variable constituted a necessary condition for the outcome variable. When the consistency index exceeds 0.90, the condition can be considered necessary for the outcome variable (Fiss, 2011). According to the analysis results in Table 10, the consistency levels of all individual condition variables and their negations did not reach the threshold for determining necessity. This result indicates that, in the formation process of digital addiction, no single factor independently serves as a necessary prerequisite, further confirming the complexity and diversity of the digital inclusion paradox. Therefore, it is necessary to incorporate the perspective of sufficient conditions in subsequent analyses to comprehensively reveal the diverse mechanisms of digital addiction formation in different contexts.

**Table 10:** Necessity test of antecedent condition variables.

Predictor variable	Consistency	Coverage
DI	0.778	0.736
~DI	0.440	0.671
IF	0.777	0.773
~IF	0.480	0.679
IR	0.772	0.774
~IR	0.490	0.685
ID	0.471	0.677
~ID	0.763	0.750
IC	0.778	0.776
~IC	0.481	0.677
IM	0.719	0.781
~IM	0.527	0.665
AP	0.749	0.767
~AP	0.508	0.691
PP	0.627	0.681
~PP	0.632	0.798
DR	0.750	0.769
~DR	0.507	0.687

A further systematic analysis was conducted on different combinations of conditional variables to identify multiple configuration paths that can form digital addiction. Through a truth table analysis that lists all possible combinations of these conditions and combines this with the sample distribution, the relationships between different configurations and the outcome variable were summarized, thus laying the foundation for the subsequent sufficiency analysis. A minimum case frequency threshold of 6 was set to exclude condition combinations with insufficient sample support and weak representativeness, ensuring that the analytical results are based on stable empirical evidence. At the same time, to guarantee the reliability of the relationship between the configurations and outcome variable, the consistency threshold and PRI threshold were set at 0.85 and 0.75, respectively. Finally, 32 genuine configurations were identified, as detailed in Table 11.

**Table 11:** Truth table summary.

DI	IF	IR	ID	IC	IM	AP	PP	DR	Number	DA
1	0	1	1	0	1	0	1	1	6	1
1	0	1	1	0	1	1	0	0	6	1
0	0	1	1	1	1	1	1	1	6	1
1	0	1	1	1	1	1	1	0	6	1
1	0	1	1	1	1	0	1	1	6	1
1	1	1	1	0	1	0	0	1	6	1
1	0	0	1	0	1	1	0	1	6	1
0	1	1	1	0	1	1	0	1	6	1
1	1	1	1	1	0	1	1	0	6	1
1	1	1	0	0	1	1	1	1	6	1
1	0	1	1	0	1	1	1	1	13	1
1	0	1	1	0	1	1	0	1	8	1
1	1	1	1	0	0	1	0	1	9	1
1	0	0	1	1	1	1	0	1	7	1
1	1	1	1	0	0	1	1	1	7	1
0	1	1	0	1	1	1	1	1	8	1
1	1	0	1	0	1	1	0	1	7	1
1	1	1	1	1	1	1	0	0	9	1
1	0	1	0	1	1	1	1	1	8	1
1	1	1	1	1	1	0	1	1	8	1
1	1	1	1	0	1	1	1	1	8	1
1	1	1	1	0	1	1	0	1	9	1
0	1	1	1	1	1	1	0	1	11	1
1	0	1	1	1	1	1	1	1	14	1
1	0	1	1	1	1	1	0	1	9	1
0	1	1	1	1	1	1	1	1	15	1
1	1	0	1	1	1	1	1	1	10	1
1	1	0	1	1	1	1	0	1	11	1
1	1	1	1	1	0	1	1	1	12	0
1	1	1	1	1	1	1	1	1	12	0
1	1	1	1	1	1	1	0	1	14	0
0	0	0	0	0	0	0	1	0	78	0

#### 5.3.3 Configuration analysis

There are seven pathways of digital addiction among the new generation of older adults, which can be categorized into three typical modes: identity operation, identity reinforcement, and norm-guided (Table 12).

**Table 12:** Configuration classification.

Prerequisite	Mode 1		Mode 2			Mode 3	
	Configuration 1a	Configuration 1b	Configuration 2a	Configuration 2b	Configuration 2c	Configuration 3a	Configuration 3b
DI	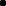	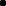	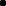	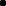	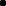		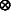
IF		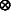		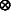	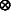	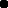	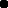
IR	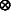		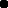	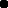	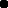	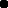	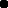
ID	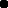	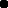	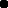	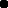	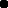		
IC			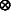		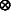	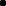	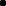
IM	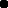	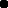				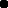	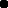
AP	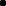	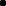	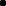	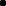	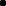		
PP	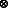	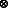			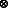	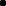	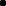
DR	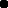	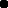					
Consistency	0.889	0.899	0.877	0.901	0.916	0.893	0.891
Raw coverage	0.221	0.217	0.229	0.229	0.178	0.205	0.187
Unique coverage	0.002	0.004	0.018	0.002	0.001	0.005	0.011
Solution consistency	0.890						
Solution coverage	0.805						

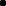
 Supplementary condition; 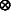 lack of core condition; 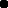 core condition; 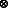 lack of auxiliary condition.

Mode one is the “Identity Operation Type”. This mode includes two typical configurations: configuration 1a is DI* ~ IR*ID*IM*AP* ~ PP*DR, and configuration 1b is DI* ~ IF*ID*IM*AP* ~ PP*DR. Digital inclusion, identity dissemination, identity maintenance, algorithmic perception, and digital reflection are the key factors influencing digital addiction among the new generation of older adults. Its core characteristic lies in the fact that, during their active process of digital inclusion to reconstruct their identities, older adults are unable to effectively transform the motives for identity dissemination and maintenance into stable self-integration. Instead, they fall into a behavioral loop of “identity operation-feedback reinforcement-deeper engagement”. Specifically, when the new generation of older adults disseminates their identities via social media, interest-based communities, and other digital settings, these efforts mutually reinforce attempts to maintain their established social identities or family roles in digital spaces. Consequently, their digital use shifts from instrumental participation to daily practices characterized by a sense of identity, responsibility, and emotional attachment. In this process, algorithmic perception is incorporated into the strategy framework of identity practices; individuals adapt to platform recommendation logic to optimize their identity presentation and interactive feedback, further increasing the frequency and intensity of digital behaviors. Although this group demonstrates a certain degree of digital reflection capability, they are unable to address the problem of the excessive centralization of digital identities. In contrast, they rationalize high-intensity and persistent usage behaviors at the cognitive level. This mode is applicable to the new generation of older adults whose social roles have significantly changed, whose digital skills are at an intermediate level, but who have a strong motivation for social expression and lack offline alternative identity support. They are more likely to regard digital spaces as the main arena for self-realization and social recognition, thereby falling into an excessively invested and hard-to-self-regulate usage pattern. These findings indicate that if the improvement of digital reflection awareness is not accompanied by behavioral guidance and support for alternative identity construction, it will be difficult to effectively curb the risk of digital addiction.

Mode Two is the “Identity Reinforcement Type”. This mode includes three typical configurations: configuration 2a is DI*IR*ID* ~ IA*AP, configuration 2b is DI* ~ IF*IR*ID*AP, and configuration 2c is DI* ~ IF*IR*ID* ~ IC*AP* ~ PP, in which digital inclusion, identity identification, identity dissemination, and algorithm perception are the key conditions influencing digital addiction among the new generation of older adults. The core feature of this mode lies in the transformation of digital space into a vital source for individuals to maintain their self-identity. As digital inclusion increases, the new generation of older adults gradually regards digital platforms as crucial arenas for defining who they are and where they belong, reinforcing their existing identities through ongoing dissemination. In this process, identity identification provides inherent legitimacy for frequent digital behaviors, while identity dissemination makes such identities explicit and reinforced through continuous interactive feedback, promoting digital usage from situational participation to a highly routinized identity practice. Simultaneously, algorithm perception enables individuals to understand and adapt to platform recommendation logic, increasing the likelihood that identity-related content is continuously pushed and affirmed. This mode mainly applies to the new generation of older adults who possess strong digital competencies, have relatively stable identities, and exhibit higher needs for social expression. Especially when real-world social interaction opportunities are limited or there is insufficient space for role fulfillment, digital spaces are more likely to become important supports for maintaining self-continuity and a sense of identity security. The above findings suggest that when digital inclusion primarily serves to confirm and reinforce identity, rather than facilitate integration and expansion, even if it appears subjectively reasonable, it may evolve into an addiction pattern that is difficult to self-regulate under the constant reinforcement of algorithmic environments.

Mode Three is the “Norm-Guided Type.” This mode includes two typical configurations: configuration 3a is IF*IR*IC*IM*PP, and configuration 3b is ~DI*IF*IR*IC*IM*PP, where identity formation, identity identification, identity co-construction, identity maintenance, and platform pressure are the key conditions influencing digital addiction among the new generation of older adults. Its core feature is that digital addiction does not stem from an individual’s active pursuit of digital inclusion but is embedded within highly socialized identity negotiation and norm-based constraints. In this pathway, the new generation of older adults gradually completes their identity formation by learning and internalizing existing behavioral norms, role expectations, and interaction rules on digital platforms, and forms relatively stable identity recognition through ongoing social interactions. However, this recognition is not entirely self-driven; rather, it is continually shaped and reinforced by external expectations, evaluations, and feedback during identity co-construction. As identity gradually solidifies, identity maintenance becomes a continuous task with normative implications; individuals need to be online frequently, respond promptly, and maintain role consistency to avoid deviating from their established identity. In this process, the platform pressure acts as a key external structural force. The implicit requirements for activity, interaction frequency, and visibility mechanisms on the platform make deviation and silence more likely to be amplified and punished, thus increasing the psychological and social costs of reducing usage or temporarily withdrawing from the platform. Consequently, the digital usage of the new generation of older adults gradually shifts from voluntary participation to normative compliance, forming a high-intensity usage pattern driven primarily by role responsibility and social expectations. This mode applies to the new generation of older adults who are highly embedded in online communities, value relationship maintenance and role stability, and are more sensitive to social evaluation. Their digital addiction is more of a structurally driven state of continuous online presence. These findings indicate that digital addiction among the new generation of older adults does not always arise from individual motivation or technological inducement but may be deeply rooted in identity norms and interaction pressures created by digital platforms. This result expands our understanding of the social origins of digital addiction among the new generation of older adults and highlights the importance of focusing on platform structures and identity expectations in future interventions.

Based on a comprehensive analysis of the three types of configuration paths, digital addiction among the new generation of older adults is shaped by multiple pathways, each activated and amplified by different identity reconstruction processes within the context of digital platforms. Pattern one presents identity-operating addiction, which centers on identity dissemination and its maintenance. It emphasizes how, under conditions of high digital inclusion and algorithmic reinforcement, incomplete identity integration is transformed into intensive digital use through ongoing identity practices. Pattern two reveals recognition-reinforcing addiction, focused on the affirmation of identity. This suggests that when digital inclusion mainly serves the stability and continuity of existing identities, algorithm-driven environments may amplify reasonable needs for recognition into persistent and difficult-to-interrupt usage patterns. Pattern three points to norm-guided addiction, associated with the joint presence of identity formulation, co-construction, and platform pressures, highlighting the structural characteristics of digital addiction in situations involving intensive social interactions and strict platform norms. Although each mode has its own emphasis, they collectively indicate that digital addiction among the new generation of older adults is more likely to occur when they actively engage in digital life and possess certain digital skills and reflective awareness. Consequently, digital platforms have shifted from being merely compensatory tools to becoming important social spaces that carry out the functions of identity construction, affirmation, and maintenance. Compared with traditional explanatory frameworks that attribute digital addiction mainly to a lack of digital skills, cognitive decline, or self-control disorders, this study reveals the differentiated identity mechanisms underlying digital addiction in the new generation of older adults. This clarifies that it is precisely the interaction between identity needs and platform structures that shapes the diverse risk patterns of digital addiction in this demographic. This finding provides a more context-sensitive theoretical basis for understanding healthy and sustainable digital inclusion.

## 6 Conclusion and implication

This study constructs a comprehensive analytical framework that integrates the relationships between digital inclusion, identity reconstruction and digital addiction to reveal the paradoxical mechanism of digital inclusion among the new generation of older adults, which aims to clarify the dynamic evolution of identity within this group throughout the process of digital inclusion and analyze their differentiated paths of digital addiction. The main research findings are as follows: (1) Digital inclusion does not directly affect digital addiction but influences the formation of sustained digital-use behaviors among the new generation of older adults by triggering and deepening multiple identity reconstruction processes. This means that the potential evolution of digital inclusion into digital addiction does not lie in technology use, but rather in the way digital practices provide the new generation of older adults with identity frameworks to redefine themselves, affirm their social status, and maintain relational connections. As digital participation becomes increasingly embedded in daily life, the new generation of older adults continuously adjusts to multiple roles, such as learner, participant, expresser, and companion. Digital inclusion has gradually evolved into an important means of maintaining identity continuity and achieving affirmation. In this process, what truly drives sustained and intense digital inclusion is not merely the accumulation of usage frequency or duration but the ongoing activation, response, and reinforcement of identity needs within the digital space. (2) Algorithmic perception, platform pressure, and digital reflection exhibit significant and differentiated moderating effects between identity reconstruction and digital addiction, highlighting the dual roles of individual cognition and platform structure in the formation of digital addiction among the new generation of older adults. This indicates that digital addiction results from the combined influence of an individual’s subjective cognitive abilities and external platform mechanisms. Specifically, the weaker the algorithmic perception, the more likely they are to regard platform recommendations, interaction rankings, and content distribution as a natural flow of information, thereby unconsciously deepening their dependence on the platform rhythm. The stronger the platform pressure, the more implicit rules, such as instant responses, constant online presence, and visible interactions, become external constraints for maintaining identity, which in turn amplifies the tension and attachment during the process of identity reconstruction. To a certain extent, digital reflection equips the new generation of older adults with the ability to recognize, assess, and interrupt this process, thereby curbing digital addiction. (3) Three types of digital addiction patterns were identified: identity operation, identity reinforcement, and norm-guided. This breaks away from traditional explanatory approaches that simply attribute digital addiction to a lack of digital skills, cognitive decline, or insufficient self-control and reveals a clear differentiation in the identity mechanisms underlying digital addiction among the new generation of older adults. It is precisely the diverse identity needs and structural forces of platforms, through their interplay, that give rise to multiple risk forms of digital addiction. This means that different types of digital addiction are not homogeneous risks; instead, each corresponds to distinct identity needs, interaction contexts, and ways of engaging with platforms. Among them, “Identity Operation Type” is more about individuals relying on digital platforms to maintain their daily roles and sense of social presence, gradually forming a path dependency through frequent use. The “Identity Reinforcement Type” mainly manifests as individuals continuously affirming their self-worth through likes, responses, displays, and being seen, making digital participation an important source of identity affirmation. The “Norm-Guided Type” originates more from the persistent pressure of platform interaction norms and relationship expectations, whereby individuals are passively drawn in due to social constraints that demand their presence and responses. These three patterns together indicate that digital addiction among the new generation of older adults is not simply an issue of ability but rather the result of an interplay between identity construction and platform governance in a digital society. In summary, from the perspective of identity reconstruction, this study deepens the understanding of the internal mechanisms underlying the paradox of digital inclusion and provides a theoretical basis and practical insights for promoting healthy and sustainable digital inclusion among the new generation of older adults. Compared to understanding the issue of digital inclusion solely from the perspectives of technical access, usage ability, or behavioral regulation, this study further reveals that only by placing digital inclusion within the interactive framework of identity reconstruction can we more accurately grasp the underlying logic behind digital addiction among the new generation of older adults and provide a more targeted analytical foundation for subsequent governance.

To guide the new generation of older adults toward sustainable and healthy digital inclusion, it is necessary to establish a multilevel system of intervention and support that reshapes the interplay between identity needs, platform structures, and social support. First, at the individual level, the focus of the intervention should shift from single-faceted digital skills training to fostering digital reflectiveness and awareness of identity boundaries. By enhancing their understanding of algorithmic recommendations, engagement incentives, and social comparison mechanisms, the new generation of older adults can strengthen their ability to recognize and regulate identity-related practices, thereby transforming intense, identity-driven digital use into a more controllable and reflective participation. Special attention should be given to helping them recognize the boundaries between self-expression and excessive involvement, as well as between maintaining relationships and being constantly online, to prevent the need for identity affirmation from continuously spilling over into dependent use without sufficient reflection. Second, at the interactional level, it is important to create a low-pressure, performance-free digital social environment by leveraging family, peer, and community networks. This involves weakening implicit norms, such as instant responses and constant connectivity, thus reducing the accumulation of social pressure that arises during mutual identity construction and maintenance and buffering against the development of norm-driven digital addiction. Family members, peer groups and community organizations, should avoid using criteria such as digital proficiency, response speed, or a constant online presence to evaluate them. Reducing the tendency to equate digital participation with proof of identity or social obligation will help preserve a more flexible and autonomous digital space for the new generation of older adults. Third, at the platform level, there should be a shift in platform design logic from usage maximization to identity-friendly approaches. By incorporating stress-reducing features into algorithms, feedback mechanisms, and incentive structures, platforms can enhance the transparency of algorithms and make usage boundaries more visible, thereby preventing needs such as identity expression and recognition from being amplified by platform mechanisms into high-risk and highly dependent usage patterns. For example, platforms can reduce the push toward “Identity Operation Type” and “Identity Reinforcement Type” by weakening continuous rewards, lowering the frequency of strong notifications, and adding prompts for the duration of use and voluntary pause options. The focus of platform governance should not only be on limiting usage time, but more importantly, on preventing platforms from amplifying individuals’ identity anxiety and relational burdens through sophisticated behavioral inducements. Fourth, at the institutional level, healthy digital inclusion should be incorporated into the overall framework of active aging and digital society governance. Through policy guidance, collaborative efforts among platform accountability, community support, and public education should be promoted to build a sustainable digital support system for the elderly, turning digital platforms from potential areas of addiction risk into important social spaces that support identity integration, social participation, and psychological wellbeing. Because different forms of risk correspond to different identity mechanisms and platform contexts, governance cannot remain at the level of a single publicity campaign or general risk reminder. Instead, a tiered and categorized support system should be established to enhance the alignment between intervention measures and risk types. In summary, addressing the paradox of digital inclusion for the new generation of older adults is not about restricting their digital participation but rather about enabling the process of identity reconstruction to unfold under conditions that are reflective and adjustable through multilevel and contextualized intervention mechanisms, thereby guiding this group toward a healthier, more inclusive, and sustainable digital inclusion pathway.

Although this study reveals the internal mechanisms by which digital inclusion influences digital addiction from the perspective of identity reconstruction and identifies different patterns of digital addiction, there is still room for further discussion regarding the transformative relationships between these types, the differentiated contextual conditions, and their long-term evolutionary processes. Furthermore, the generalizability of these findings across broader samples, different platform contexts, and various social relationship structures still needs to be tested in future studies. Future research could further combine longitudinal tracking data or in-depth qualitative materials to uncover the dynamic evolutionary mechanisms of identity reconstruction as it progresses through the life course and accumulates digital experiences. Additionally, expanding the theoretical framework proposed in this study to encompass broader contexts, such as urban–rural differences, family structures, and platform types, will continue to enrich the understanding of the paradox of digital inclusion among the new generation of older adults.

## Data Availability

The datasets generated during and analyzed during the current study are available from the corresponding author upon reasonable request. Requests to access these datasets should be directed to Haibei Chen, haibeichen17@163.com.
